# Coronavirus Spike Protein Inhibits Host Cell Translation by Interaction with eIF3f

**DOI:** 10.1371/journal.pone.0001494

**Published:** 2008-01-30

**Authors:** Han Xiao, Ling Hui Xu, Yoshiyuki Yamada, Ding Xiang Liu

**Affiliations:** Institute of Molecular and Cell Biology, Proteos, Singapore; Karolinska Institutet, Sweden

## Abstract

In response to viral infection, the expression of numerous host genes, including predominantly a number of proinflammatory cytokines and chemokines, is usually up-regulated at both transcriptional and translational levels. It was noted that in cells infected with coronavirus, transcription and translation of some of these genes were differentially induced. Drastic induction of their expression at the transcriptional level was observed in cells infected with coronavirus. However, induction of the same genes at the translational level was usually found to be minimal to moderate. To investigate the underlying mechanisms, yeast two-hybrid screen was carried out using SARS-CoV proteins as baits, revealing that a subunit of the eukaryotic initiation factor 3 (eIF3), eIF3f, may interact with the N-terminal region of the SARS-CoV spike (S) protein. This interaction was subsequently confirmed by co-immunoprecipitation and immunofluorescent staining. Meanwhile, parallel experiments confirmed that eIF3f could also interact with the S protein of another coronavirus, the avian coronavirus infectious bronchitis virus (IBV). These interactions led to the inhibition of translation of a reporter gene in both in vitro expression system and intact cells. Interestingly, IBV-infected cells stably expressing a Flag-tagged eIF3f showed much higher translation of IL-6 and IL-8, suggesting that the interaction between coronavirus S protein and eIF3f plays a functional role in controlling the expression of host genes, especially genes that are induced during coronavirus infection cycles. This study reveals a novel mechanism exploited by coronavirus to regulate viral pathogenesis.

## Introduction

Activation and induction of host gene expression at both transcriptional and translational levels by virus infection constitute essential steps in host anti-viral response and viral pathogenesis. As one of the major factors that causes tissue damage and viral pathogenesis, induction and over-production of proinflammatory cytokines and chemokines are a common phenomenon in many viral infections [1]–[6]. However, it was frequently observed that the expression of these genes was differentially up-regulated at the transcriptional and translational levels in cells infected with a certain virus. For example, in cells infected with severe acute respiratory syndrome coronavirus (SARS-CoV) and in sera from SARS patients, significant up-regulation of the transcription of proinflammatory cytokines and chemokines, such as CCL1, CCL2, CCL3, CCL5, CCL9, CXCL10, IL-6, IL10 and IL12, was reported [5], [7]–[10]. However, only a moderate increase of the expression of these genes at the protein level was detected [8]–[9]. This differential up-regulation of the host gene expression at transcriptional and translational levels may simply reflect the relative capacity of host transcription and translation machinery in the infected cells. Alternatively, it would consist of a convenient and clever viral strategy to counteract host cell anti-viral response, as over-production of these host proteins is usually harmful to viruses. In this study, we show a novel mechanism exploited by coronavirus to regulate the translation of virus-induced genes at late stages of the virus infection cycle.

Coronavirus is an important pathogen of human and animals. It is the etiological agent of SARS [11]–[12]. Coronavirus is an enveloped virus with a single strand, positive-sense RNA genome of 27–32 kb in length. In coronavirus-infected cells, a 3′-coterminal nested set of six to nine mRNA species, including the genome-length mRNA (mRNA1) and five to eight subgenomic mRNA species (mRNA2-9), is expressed. The genome-length mRNA1 encodes two overlapping replicase proteins in the form of polyproteins 1a and 1a/b, which are processed by virus-encoded proteinases into at least 16 putative nonstructural proteins (NSP1-NSP16) [13]–[15]. The four major structural proteins, spike (S), envelope (E), membrane (M) and nucleocapsid (N), are encoded by different subgenomic mRNAs. In addition, several accessory proteins are also encoded by subgenomic mRNAs [15]. In coronavirus-infected cells, no obvious inhibition of host protein synthesis was observed at least at early stages of the virus infection cycle.

Viruses rely on the canonical cellular translation machinery to translate their own RNAs. This would facilitate the rapid production of viral proteins and meanwhile, render inhibitory effect on the production of host proteins including host anti-viral proteins [16]. In fact, viruses may inhibit host protein synthesis by targeting multiple steps in the gene expression process via various vices. For instance, vesicular stomatitis virus M protein inhibits the initiation of transcription of host genes [17]–[20], while adenovirus encodes VA RNA, a small highly structured RNA that competitively binds to the dsRNA-binding site of the double-strand RNA activated kinase (PKR) and prevents its activation [21]. The majority of control over cellular mRNA translation, however, occurs at the initiation stage, the rate-limiting step of protein synthesis for many RNA viruses [22]–[23]. Translation initiation begins with binding of the initiator Met-tRNA_i_ to the 40S ribosomal subunit. This is facilitated by the formation of a eukaryotic initiation factor 2 (eIF2)/GTP/Met-tRNA_i_ ternary complex and a subsequent 43S preinitiation complex. Viruses could inhibit cellular protein synthesis by targeting and inactivating components of the initiation complex. One well studied example is the picornaviral 2A proteinase which cleaves the initiation factor eIF4G within its N-terminal region containing the binding site for eIF4E and poly(A)-binding protein (PABP) [24]–[27]. The cleaved C-terminal region of eIF4G could be associated with eIF3 and eFI4A, binds with higher affinity to viral mRNAs and facilitates translation of its own protein [26], [28].

The initiation factor eIF3 plays an important role during translation initiation by bridging the 43S preinitiation complex to mRNA via the cap binding complex eIF4F. Assembled into a large multi-subunit protein complex of approximately 650 kDa, eIF3 consists of at least 10 subunits, p170, p166, p110, p66, p48 (int6), p47 (eIF3f), p44, p40, p36 and p35 [29]–[31]. We show here that eIF3f could interact with the S protein from two coronaviruses, SARS-CoV and the coronavirus infectious bronchitis virus (IBV). These interactions led to the inhibition of translation. Interestingly, IBV-infected cells stably expressing a Flag-tagged eIF3f showed much higher translation of IL-6 and IL-8. This study reveals a novel mechanism exploited by coronavirus to counteract host-antiviral response and, meanwhile, regulate the pathogenesis of coronavirus.

## Materials and Methods

### Cells and Cell Culture

Cells were cultured in complete Dulbecco's modified Eagle's medium (DMEM) supplemented with 5% newborn calf serum (Sterile) and 1% penicillin/streptomycin (Invitrogen), and maintained at 37°C in humidified 5% CO_2_.

### Yeast two-hybrid Screening

The N-terminal region from amino acids 20 to 404 (S**Δ**C) of the SARS-CoV S protein was used as bait to screen a cDNA library prepared from HeLa cells (BD Biosciences Clontech), as previously described [32]. Plasmid pGBKT7 contains an ADH1 promoter which directs high level expression of fusion protein in yeast; it also contains a T7 promoter and a c-Myc epitope tag. Briefly, the bait construct pGBKT7-S**Δ**C was first transformed into the yeast strain AH109 using lithium acetate method described in the Clontech manual, and 100 µg of cDNA library DNA was sequentially transformed into the pGBKT7-S**Δ**C-transformants. The transformed cells were plated on minimal selective synthetic dropout (SD) media SD/-Leu/-Trp/-His plate and positive colonies were selected by dotting colonies onto SD/-Leu/-Trp/-His/-X-α-gal plates. Potential positive gene was determined by direct PCR of individual yeast colonies followed by automated nucleotide sequencing.

### Co-immunoprecipitation and Western blot

Exponentially growing cells were seeded at ∼2×10^6^ in 6 well plates and transfected with appropriate plasmids. At 24 hours post-transfection, the cells were washed with ice-cold PBS twice and lysed with 250 µl of ice-cold cell lysis buffer (140 mM NaCl, 10 mM Tris-HCl pH 8.0 and 5 mM NP-40). The total cell lysates were clarified by centrifugation at 13,000 rpm for 10 minutes, and the first antibody was added to the supernatants. After incubation at room temperature for 45 minutes, protein A-Sepharose beads were added, and incubation was continued for an additional 30 min. The precipitates were collected by centrifugation and the beads were washed 5 times with the lysis buffer before subjected to SDS-PAGE. In some cases, M2 anti-Flag Sepharose beads (Sigma), instead of the primary antibodies and protein A-Sepharose beads, were used.

### Purification of recombinant protein from E. coli

Plasmids pGEX-5X1, pGEX-S**Δ**C and pGEX-3f were expressed in bacteria by induction with 0.4 mM IPTG at 37°C for 3 hours. GST and GST fusion protein were purified using the GST purification module (Amersham).

### Sucrose density gradient centrifugation

15%–50% linear sucrose gradients in PBS with protease inhibitor cocktail (Roche) were prepared using a Hoefer SG15 gradient maker. The gradients were allowed to stand for 3 to 5 hours at 4°C before layering a 0.2-ml sample onto a gradient. The GST-3f and GST-S**Δ**C proteins were purified using the GST purification module (Amersham), and 10 µg of each protein were loaded onto the top of the gradient and centrifuged in a Beckman LZ-65 ultracentrifuge using an SW60 rotor at 40,000 g for 20 hours. After centrifugation, 14 fractions were collected from the top to the bottom of the gradient and analyzed by Western blot.

### In vitro transcription and translation inhibition assay

In vitro transcription of luciferase RNA was preformed using T7 RNA polymerase (Promega). After extraction with phenol/chloroform, the in vitro transcribed RNA was precipitated with ethanol and dissolved in RNase-free water.

In vitro translation was performed in rabbit reticulocyte lysates in the presence of [^35^S] methionine based on the protocol recommended by the manufacturer (Promega). The in vitro transcribed Luc mRNA was added to a master tube containing the in vitro translation reaction mixture. The mixture was then aliquoted and increasing amounts of purified GST and GST-S**Δ**C proteins were added to each tube as indicated. Translation was allowed to proceed at 30°C for 90 min. After incubation, a 5 µl aliquot of the reaction was resolved by SDS-10% polyacrylamide gel and the polypeptides were detected by autoradiography.

### Transient expression of viral protein in mammalian cells

Viral or cellular genes cloned into plasmids under the control of a T7 promoter were transiently expressed in mammalian cells using a vaccinia virus/T7 system, as previously described [33]. Briefly, semiconfluent monolayers of HeLa cells were infected with a recombinant vaccinia virus (vTF7-3), which expresses the T7 RNA polymerase gene, for 2 hours at 37°C prior to transfection. The plasmid DNA was transfected into vTF7-3-infected cells using Effectene transfection reagent (QIAGEN) according to the manufacturer's instructions.

### Inhibition assay in intact cells

HeLa cells on 6-well plates were infected with the recombinant vaccinia virus vTF7-3, and transfected with 1.2 µg of empty vector pKT0, 1 µg pKT0/S+0.2 µg pLUC, and 1 µg pKT0+0.2 µg pLUC, respectively, using the effectene transfection kit (QIAGEN). At 24 hours post-transfection, cells were lysed using the passive cell lysis buffer provided by Promega and luciferase activity was measured using the Dual-Luciferase Reporter Assay System (Promega).

### Immunofluorescence

HeLa cells, grown on 24 well plates, were infected with vTF7-3 and transfected with appropriate plasmid DNA. After washing with PBS, the cells were fixed with 4% paraformaldehyde for 30 minutes at room temperature and permeabilized with 0.2% Triton X-100, followed by incubation with specific antiserum at room temperature for 2 hours. The cells were then washed with PBS and incubated with fluorescein isothiocyanate- or tetramethylrhodamine isothiocyanate-conjugated anti-rabbit or anti-mouse IgG (Sigma) in fluorescence dilution buffer at 4°C for 2 hours before mounting.

### Construction of plasmids

Plasmid pGBKT7-S**Δ**C (denoted as S**Δ**C), which covers the N-terminal region (20-404aa) of the SARS-CoV S protein, was constructed by cloning an EcoRI/BamHI digested PCR fragment into EcoRI/BamHI digested pGBKT7 (Clontech). The PCR fragment was generated with full-length SARS-CoV S gene using primers (5′-CGGAATTCCTTGACCGGTGCACC ACTT-3′) and (5′-CGGGATCCCTGGCGCTATTTGTCTTACATC-3′). Plasmid pIBV-S**Δ**C, which covers the equivalent region to the S**Δ**C of SARS-CoV, was PCR amplified using specific primers pairs and cloned into pGBKT7. Plasmid pFlag-3f was created by cloning a SmaI/HindIII digested pGAD-3f fragment into EcoRV/HindIII digested pFlag. Plasmid pGEX-S**Δ**C was created by cloning a EcoRI/SalI digested pGBKT7-S**Δ**C into EcoRI/XhoI digested pGEX-5X1. Plasmid pGEX-3f was constructed by cloning a EcoRI/XhoI digested pGAD-3f into EcoRI/XhoI digested pGEX-5X3. All constructs were confirmed by automated nucleotide sequencing.

## Results

### Interaction of SARS-CoV S protein with eIF3f

**Figure 1:**
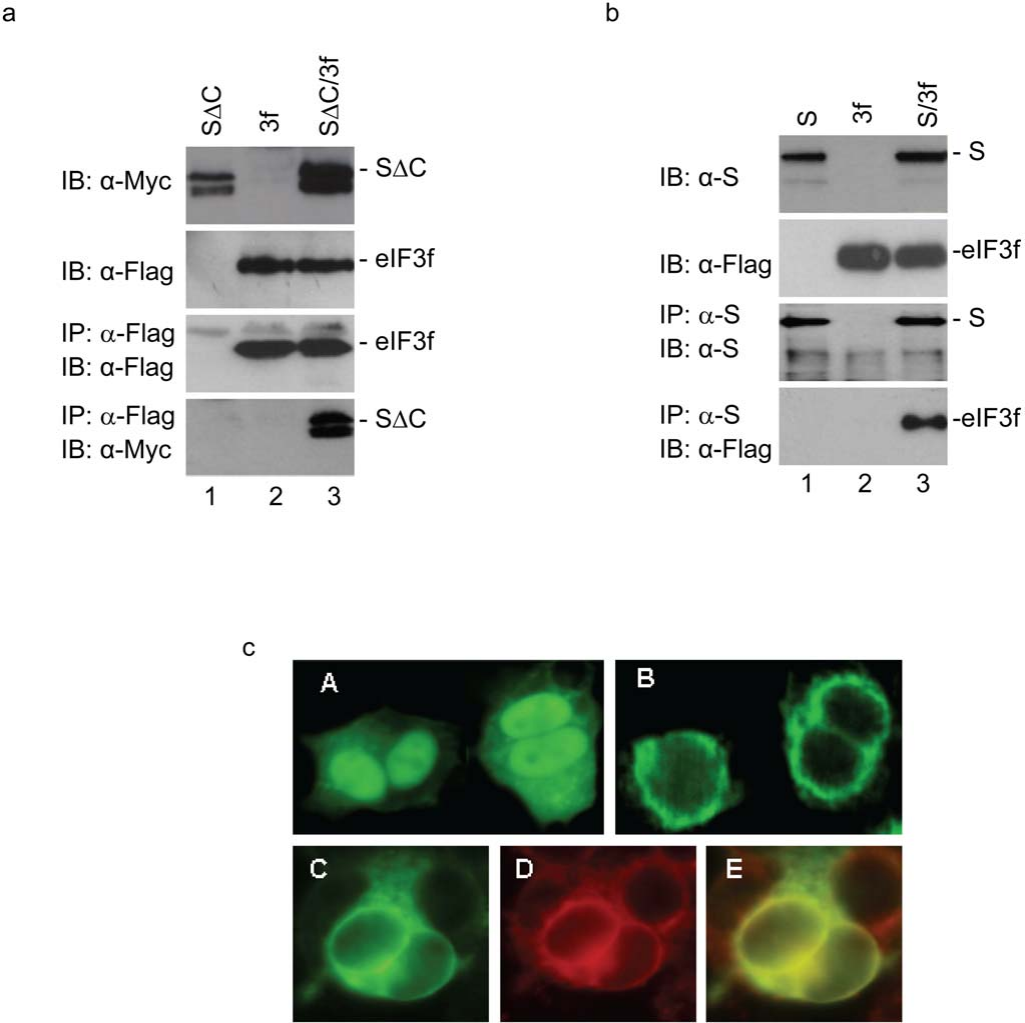
Confirmation of the interaction between SARS-CoV S protein and eIF3f by co-immunoprecipitation and immunofluorescence. a. Interaction of eIF3f with the N-terminal portion of SARS-CoV S protein (SΔC). HeLa cells expressing the Myc-tagged SΔC (lane 1), the Flag-tagged eIF3f (lane 2) and eIF3f+SΔC (lane 3) were harvested at 24 hours post-transfection and lysed. The total lysates were either detected directly by Western blot with anti-Myc (top panel) and anti-Flag (second panel) antibodies, respectively, or immunoprecipitated with anti-Flag antibody. The precipitates were analyzed by Western blot with anti-Flag (third panel) and anti-Myc (bottom panel) antibodies. b. Interaction of eIF3f with the full-length SARS-CoV S protein. HeLa cells expressing the SARS-CoV S (lane 1), the Flag-tagged eIF3f (lane 2) and eIF3f+S (lane 3), were lysed. The total lysates were either detected directly by Western blot with anti-S (top panel) and anti-Flag (second panel) antibodies, respectively, or immunoprecipitated with anti-S antibodies. The precipitates were analyzed by Western blot with anti-S (third panel) and anti-Flag (bottom panel) antibodies. c. Subcellular localization of eIF3f and SARS-CoV S protein by immunofluorescent staining of HeLa cells expressing eIF3f (panel A), SARS-CoV S protein (panel B) or co-expressing eIF3f and S (panels C, D and E). The subcellular localization of the proteins were examined at 12 hours post-transfection by dual labelling with a mixture of anti-S (rabbit) and anti-Flag (mouse) antibodies, followed by incubating with a mixture of FITC-conjugated anti-mouse and tetramethyl rhodamine isocyanate-conjugated anti-rabbit antisera. Panel C shows the staining profile of eIF3f, panel D shows the staining pattern of SARS-CoV S protein, and panels E shows the overlapping images C and D.

Virus infection activates transcription and translation of many host genes. During the course of studying the gene expression profiles in cells infected with coronaviruses, it was noted that drastic induction of many virus-inducible genes occurred at the transcriptional level. However, induction of the same genes at the translational level was found to be usually minimal to moderate. To search for cellular proteins that are involved in protein synthesis and may interact with coronavirus proteins, yeast two-hybrid screening was carried out using different coronavirus proteins as baits. This screening led to the identification of eIF3f that could potentially interact with the N-terminal portion (S**Δ**C) of the SARS-CoV S protein. Co-immunoprecipitation experiments were performed to further test the interaction between S**Δ**C and eIF3f. To facilitate detection of the two proteins, S**Δ**C was tagged with a Myc tag and eIF3f with a Flag tag at the N-termini, respectively. Analysis of HeLa cells expressing the Myc-tagged S**Δ**C either on its own or together with the Flag-tagged eIF3f by Western blot with anti-Myc monoclonal antibody led to efficient detection of the Myc-tagged S**Δ**C (Fig. 1a, top panel, lanes 1 and 3). Similarly, analysis of cells expressing the Flag-tagged eIF3f either on its own or together with the Myc-tagged S**Δ**C by Western blot with anti-Flag monoclonal antibody showed presence of the Flag-tagged eIF3f (Fig. 1a, second panel, lanes 2 and 3). The same cell lysates were then subjected to immunoprecipitation with anti-Flag antibody. Western blot analysis of the precipitates with anti-Flag antibody detected the Flag-tagged eIF3f expressed either on its own or together with the Myc-tagged S**Δ**C (Fig. 1a, third panel, lanes 2 and 3). Western blot analysis of the same precipitates with anti-Myc antibody showed presence of the Myc-tagged S**Δ**C only in cells co-expressing the two proteins (Fig. 1a, bottom panel, lane 3). These results confirm that the N-terminal portion of the SARS-CoV S protein can indeed interact with eIF3f.

Similar experiments were then performed to check if the full-length SARS-CoV S protein could also interact with eIF3f. As shown in Fig. 1b, efficient detection of the full-length S protein was obtained in cells expressing S protein either on its own or together with the Flag-tagged eIF3f by Western blot with anti-S polyclonal antibodies (Fig. 1b, top panel, lanes 1 and 3). Analysis of cells expressing the Flag-tagged eIF3f either on its own or together with S protein by Western blot with anti-Flag monoclonal antibody showed presence of the Flag-tagged eIF3f (Fig. 1b, second panel, lanes 2 and 3). Immunoprecipitation of the cell lysates with anti-S antibodies and subsequent analysis of the precipitates by Western blot with anti-S antibodies detected the S protein in cells expressing the protein either on its own or together with the Flag-tagged eIF3f (Fig. 1b, third panel, lanes 1 and 3). Western blot analysis of the same precipitates with anti-Flag antibody showed presence of the Flag-tagged eIF3f only in cells co-expressing the two proteins (Fig. 1b, bottom panel, lane 3). These results confirm that the full-length S protein can also interact with eIF3f.

This interaction was further investigated in cells co-expressing the two proteins by immunofluorescent staining. In HeLa cells expressing the Flag-tagged eIF3f alone, the protein was located in both the cytoplasm and the nucleus with predominant nuclear localization in some cells (Fig. 1c, panel A). In cells expressing the S protein alone, the protein is exclusively detected in the cytoplasm (Fig. 1c, panel B). In cells co-expressing the two proteins, the Flag-tagged eIF3f was found to exhibit a subcellular localization pattern similar to the S protein (Fig. 1c, panels C–E). The staining patterns of the two proteins are well overlapped (Fig. 1c, panel E), suggesting that the S protein may sequester the eIF3f to the cytoplasm.

### In vitro formation of complexes between GST-SΔC and GST-eIF3f

**Figure 2:**
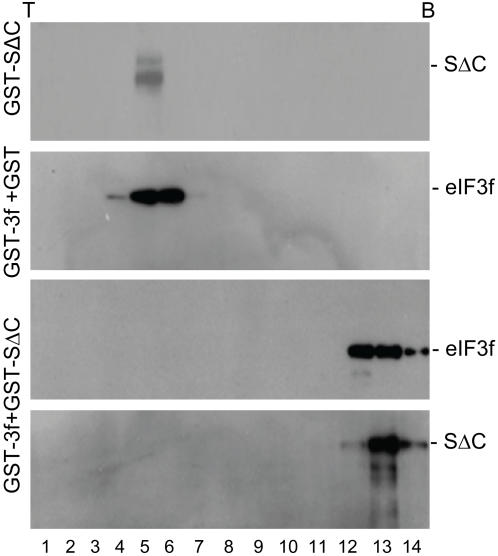
Co-fractionation of SΔC with eIF3f in vitro in sucrose gradients. Ten micrograms each of the purified GST-SΔC (top panel), GST-3f+GST (second panel) and GST3f+GST-SΔC (third and bottom panels) were loaded onto the top of 15–50% sucrose gradients and centrifuged at 40,000 g for 20 hours. Fourteen fractions were collected from the top to the bottom and analyzed by Western blot with anti-S (top and bottom panels) and anti-eIF3f (second and third panels) antibodies.

The interaction between SARS-CoV S protein and eIF3f was further investigated by checking if the two proteins could form complexes in vitro. The S**Δ**C and eIF3f were expressed in E. coli as GST fusion proteins and purified to near homogeneity (data not shown). The purified proteins GST-S**Δ**C either on its own or mixed with an equal amount of GST-eIF3f was subjected to ultracentrifugation through 15–50% sucrose gradients, and 14 fractions were collected. As shown in Fig. 2, GST-S**Δ**C on its own was exclusively detected in fraction 5 (top panel). When GST-eIF3f was mixed with the purified GST alone, it was mainly detected in fractions 4–6 (Fig. 2, second panel). When GST-eIF3f was mixed with GST-S**Δ**C, both proteins were detected in fractions 11–14 (Fig. 2, third and bottom panels). These results substantiate the conclusion that S**Δ**C could interact with eIF3f and further demonstrate that the two proteins could form stable complexes *in vitro.*

### Inhibition of the translation of a reporter gene by SARS-CoV S protein

**Figure 3:**
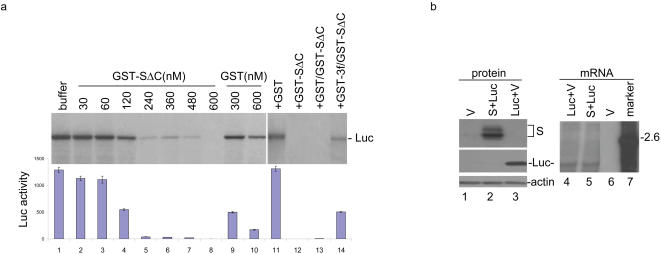
Inhibition of protein synthesis by interaction of SARS-CoV S protein with eIF3f. a. Dose dependent inhibition of luciferase mRNA translation by GST-SΔC in vitro (lanes 1–10) and rescue of the inhibition by addition of the purified GST-3f (lanes 11–14). In vitro transcribed luciferase mRNA (0.5 µg) was translated *in vitro* in rabbit reticulocyte lysates in the presence of [^35^S] methionine. Glutathione buffer (lane 1), increasing amounts of GST-SΔC (lanes 2–8), GST (lanes 9–10), purified GST (lane 11), GST-SΔC (lane 12), GST+GST-SΔC (lane 13), or GST-SΔC+GST-3f (lane 14) were added to the translation system as indicated. Polypeptides were separated on SDS-10% polyacrylamide gel and visualized by autoradiography (upper panels). A duplicate of the in vitro translation reactions in the absence of [^35^S] methionine (replaced by cold-methionine) was used to measure the luciferase activity (lower graph). b. Inhibition of luciferase translation by the full-length SARS-CoV S protein in intact cells. HeLa cells on 6-well plates were infected in duplicate with the recombinant vaccinia virus vTF7-3, and transfected with 1.2 µg of empty vector pKT0 (V) (lanes 1 and 6), 1 µg pKT0/S+0.2 µg pLUC (lanes 2 and 5), and 1 µg pKT0+0.2 µg pLUC (lanes 3 and 4), respectively. Cells were harvested at 24 hours post-transfection, and protein expression was analyzed by Western blot with anti-luciferase and anti-S antibodies. The same membrane was also probed with anti-actin antibodies as a loading control. The luciferase gene expression at the mRNA level in the transfected cells was also analyzed by Northern blot with a Dig-labeled DNA probe corresponding to the 5′-end 550 nucleotides of the luciferase gene (lanes 4–7).

We next set up to study the functional consequence of the confirmed interaction between SARS-CoV S protein and eIF3f in vitro. Equal amounts of the in vitro transcribed mRNA derived from the luciferase gene were translated in the presence of increasing amounts of purified GST-S**Δ**C protein. As shown in Fig. 3a, progressive inhibition of the luciferase gene expression was observed when 30 to 600 nM of GST-S**Δ**C protein was added to the system (lanes 2–8). Complete inhibition of the luciferase expression was achieved when 600 nM of the purified GST-S**Δ**C protein was used (Fig. 3a, lane 8). In contrast, much less inhibitory effect was observed when 300 and 600 nM of purified GST was used (Fig. 3a, lanes 9 and 10).

As 600 nM of GST-S**Δ**C was shown to completely inhibit the expression of luciferase, addition of equal amount of the purified GST-eIF3f to the in vitro translation system was carried out to see if the inhibitory effect can be reversed. As anticipated, addition of exogenous GST-eIF3f fusion protein substantially relieved the inhibitory effect (Fig.3a, lane 14). However, addition of the same amount of the purified GST did not rescue the expression of the reporter gene (Fig. 3a, lane 13).

To further test if similar inhibition of the reporter gene translation could be observed in intact cells, luciferase gene under the control of a minimal promoter was co-transfected into HeLa cells either with an empty control plasmid (pKT0) or the full-length S protein. At 24 hours post transfection, cells were lysed and aliquoted. A portion of the lysates was used for luciferase assay while another was used for Western blot analysis of the protein expression. Expression of S protein could substantially inhibit the luciferase expression (Fig.3b, lane 2), whereas the empty vector alone did not cause any inhibition (Fig. 3b, lane 3). Quantitative analysis of the inhibitory effect by measuring the luciferase activity showed that in cells transfected with Luc+empty vector, the relative luciferase activity is 1936 light units, which are 37-fold higher than that in cells transfected with Luc+S. Northern blot analysis confirmed that approximately equal amounts of luciferase mRNA were detected in cells transfected with luciferase together either with an empty vector or the S construct (Fig. 3b, lanes 4 and 5).

### Interaction of IBV S protein with eIF3f and inhibition of the translation of a reporter gene

**Figure 4:**
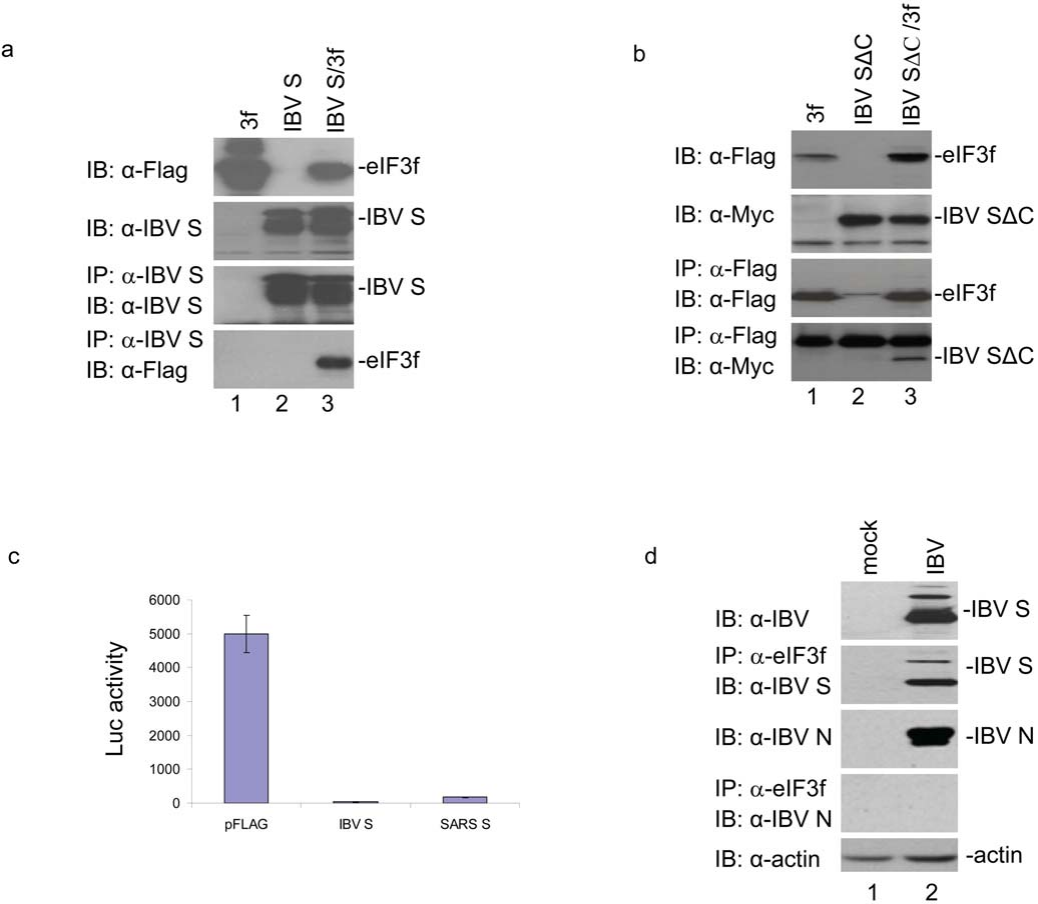
Interaction of the IBV S protein with eIF3f and inhibition of protein synthesis. a. Confirmation of the interaction between the Flag-tagged eIF3f and the full-length IBV S protein by co-immunoprecipitation. HeLa cells expressing the Flag-tagged eIF3f (lane 1), IBV S (lane 2) and eIF3f+IBV S (lane 3) by using the vaccinia/T7 expression system were harvested at 24 hours post-transfection and lysed. The total cell lysates were either detected directly by Western blot with anti-Flag (top panel) and anti-IBV S (second panel) antibodies or immunoprecipitated with anti-IBV S antibodies. The precipitates were analyzed by Western blot with anti-IBV S (third panel) and anti-Flag (bottom panel) antibodies. b. Interaction of the Flag-tagged eIF3f with the N-terminal region of the IBV S protein (IBV SΔC). HeLa cells expressing the Flag-tagged eIF3f (lane 1), the Myc-tagged IBV SΔC (lane 2) and eIF3f+IBV SΔC (lane 3), were harvested at 24 hours post-transfection and lysed. The total cells lysates were either detected directly by Western blot with anti-Flag (top panel) and anti-Myc (second panel) antibodies, or immunoprecipitated with anti-Flag antibody. The precipitates were analyzed by Western blot with anti-Flag (third panel) or anti-Myc (bottom panel) antibodies. c. Inhibition of luciferase activity by IBV and SARS-CoV S protein. HeLa cells expressing luciferase, luciferase+IBV S and luciferase+SARS-CoV S by using the vaccinia/T7 system were harvested at 24 hours post-transfection, lysed, and the luciferase activities in the total cell lysates were measured. d. Co-immunoprecipitation analysis of the interaction between IBV S protein and the endogenous eIF3f in IBV-infected Vero cells. Mock- and IBV-infected Vero cells were harvested at 24 hours post-infection and lysed. The total cell lysates were either detected directly by Western blot with anti-IBV S (top panel), anti-IBV N (third panel) and anti-actin (bottom panel) antibodies, respectively, or immunoprecipitated with anti-eIF3f antibody. The precipitates were analyzed by Western blot with anti-IBV S (second panel) or N (fourth panel) antibodies.

Co-immunoprecipitation was performed to check if IBV S protein could also interact with eIF3f. Detection of the full-length IBV S protein was obtained in cells expressing S protein either on its own or together with the Flag-tagged eIF3f by Western blot analysis with anti-IBV S polyclonal antibodies (Fig. 4a, second panel, lanes 2 and 3). Analysis of cells expressing the Flag-tagged eIF3f either on its own or together with S protein by Western blot with anti-Flag monoclonal antibody showed presence of the Flag-tagged eIF3f (Fig. 4a, top panel, lanes 1 and 3). Immunoprecipitation of the cell lysates with anti-IBV S antibodies and subsequent analysis of precipitates by Western blot with anti-IBV S antibodies detected the S protein in cells expressing the protein either on its own or together with the Flag-tagged eIF3f (Fig. 4a, third panel, lanes 2 and 3). Western blot analysis of the same precipitates with anti-Flag antibody led to the detection of the Flag-tagged eIF3f only in cells co-expressing the two proteins (Fig. 4a, bottom panel, lane 3). These results confirm that the full-length IBV S protein could also interact with eIF3f.

Co-immunoprecipitation experiments were then performed to check if the N-terminal region of IBV S protein equivalent to the S**Δ**C of SARS-CoV S protein could interact with eIF3f. Similarly, the IBV S**Δ**C was tagged with the Myc tag and eIF3f with the Flag tag at the N-termini, respectively. Analysis of cells expressing the Myc-tagged IBV S**Δ**C either on its own or together with the Flag-tagged eIF3f by Western blot with anti-Myc monoclonal antibody showed presence of the Myc-tagged S**Δ**C (Fig. 4b, second panel, lanes 2 and 3). Similarly, analysis of cells expressing the Flag-tagged eIF3f either on its own or together with the Myc-tagged IBV S**Δ**C by Western blot with anti-Flag monoclonal antibody detected the Flag-tagged eIF3f(Fig. 4b, top panel, lanes 1 and 3). Immunoprecipitation of the same cell lysates with anti-Flag antibody and subsequent analysis of the precipitates by Western blot with anti-Flag antibody led to the detection of the Flag-tagged eIF3f expressed either on its own or together with the Myc-tagged IBV S**Δ**C (Fig. 4b, third panel, lanes 1 and 3). Western blot analysis of the same precipitates with anti-Myc antibody detected the Myc-tagged S**Δ**C only in cells co-expressing the two proteins (Fig. 4b, bottom panel, lane 3). These results confirm that the N-terminal portion of the IBV S protein can also interact with eIF3f.

Measurement of the luciferase activity in cells co-transfected with either IBV or SARS CoV S protein showed that the luciferase activity was drastically reduced (Fig. 4c). It confirms that IBV S protein could also inhibit translation of the reporter gene.

Co-immunoprecipitation experiments were then carried out to test if IBV S protein could interact with the endogenous eIF3f in IBV-infected cells. As shown in Fig. 4d, Western blot analysis showed presence of the S and N proteins in IBV-infected cells using anti-IBV S and N antibodies, respectively (top and third panels). Immunoprecipitaion of the same lysates with anti-eIF3f antibodies and subsequent analysis of the precipitates by Western blot with anti-IBV S antibodies led to the detection of the S protein in IBV-infected but not in mock-infected cells (Fig. 4d, second panel). Western blot analysis of the same precipitates with anti-IBV N antibodies, however, did not detect the N protein (Fig. 4d, fourth panel). These results confirm that IBV S could indeed interact with the endogenous eIF3f protein in virus-infected cells.

### Significantly more efficient translation of proteins in cells expressing a fusion-competent S protein than in cells expressing a fusion incompetent S protein

**Figure 5:**
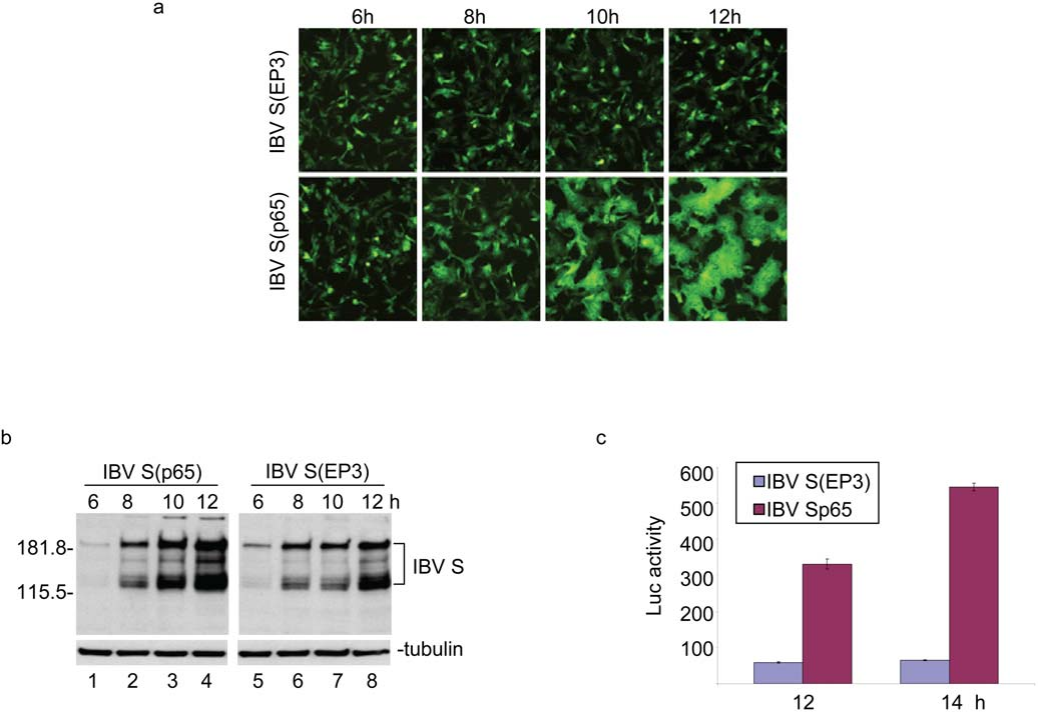
The relationship between the fusogenicity of S protein and its expression level in cells expressing fusion-competent and fusion-incompetent S protein. a. Detection of cell-cell fusion by indirect immunofluorescence. Vero cells were infected with the vaccinia/T7 recombinant virus and transfected with S(EP3) and S(p65) constructs. At 6, 8, 10 and 12 hours post-transfecion, respectively, cells were fixed with 4% paraformaldehyde and stained with rabbit anti-IBV S polyclonal antibodies. b. Expression of fusion-competent and fusion-incompetent IBV S protein. Vero cells were infected with vaccinia/T7 recombinant virus and transfected with S(EP3) and S(p65) constructs. Cells were harvested at 6, 8, 10 and 12 hours post-transfection, respectively, and lysates prepared. The viral protein expression was analyzed by Western blot with rabbit anti-IBV S antibodies. The same membrane was also probed with anti-β-tubulin monoclonal antibody as a loading control. c. Quantitative analysis of the expression of fusion-competent and fusion-incompetent IBV S protein. Vero cells were infected with vaccinia/T7 recombinant virus and transfected with S(EP3)-luciferase and S(p65)-luciferase constructs. Cells were harvested at 12 and 14 hours post-transfection, respectively, lysates prepared, and the luciferase activity was determined.

Expression in cells of a fusion-competent IBV S protein (IBV S(p65)) cloned from a Vero cell-adapted IBV passage 65 (p65) and fusion-incompetent S protein (IBV S(EP3)) cloned from chicken embryo passage 3 (EP3) (unpublished observation) was carried out to support that interaction between S protein and eIF3.5 leads to inhibition of protein translation. It was reasoned that in cells transfected with IBV S(EP3), expression of the S protein would be inhibited after the protein synthesis reaches to a certain level, as the S protein already synthesized would bind to eIF3.5, leading to the inhibition of translation and a lower level of S protein synthesis. On the contrary, in cells transfected with IBV S(p65), these initially transfected cells would fuse with the neighboring cells after the S protein expression reaches to a certain level and the eIF3.5 factor present in these neighboring cells would offset the inhibitory effect of the S protein, resulting in a higher level of S protein synthesis. As expected, no cell-cell fusion was observed in cells expressing IBV S(EP3) at 6, to 12 hours post-infection (Fig. 5a). In cells expressing IBV S(p65), small syncytial cells were observed at 8 hours post-infection (Fig. 5a). Giant syncytial cells were observed on almost the whole monolayer at 10 to 12 hours post-infection (Fig. 5a). Western blot analysis showed that slightly more S protein was observed in cells expressing S(EP3) construct at 6 hours post-infected, compared to cells expressing S(p65) construct (Fig. 5b, lanes 1 and 5). At 8 hours post-infection, approximately equal amounts of S protein were detected in cells expressing both constructs (Fig. 5b, lanes 2 and 6). Much more S protein was detected in cells expressing S(p65) than did in cells expressing S(EP3) at 10 and 12 hours post-infection (Fig. 5b, lanes 3, 4, 7 and 8). As a loading control, very similar amounts of the β-tubulin were detected in samples (Fig. 5b). Analysis of the expression of the two constructs at the mRNA level by realtime RT-PCR showed that very similar amounts of mRNA were detected in cells expressing the two constructs at 6–10 hours post-transfection.

To obtain more quantitative data, the luciferase gene was fused to the C-terminus of the S(EP3) and S(p65), respectively. A furin cleavage site was placed between the S and luciferase sequences. As shown in Fig. 5c, transfection of the two constructs into Vero cells showed presence of 5 and 10 fold more luciferase activity in cells expressing the S(p65)-luciferase fusion construct than in cells expressing the S(EP3)-luciferase construct at 12 and 14 hours post-transfection, respectively (Fig. 5c).

### Enhanced translation of IL-6 and IL-8 in IBV-infected cells stably expressing the Flag-tagged eIF3f

**Figure 6:**
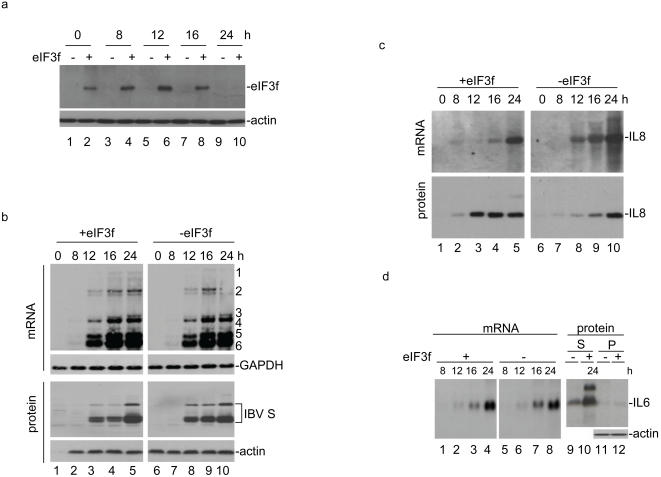
Analysis of IL-6 and IL-8 expression at mRNA and protein levels in IBV-infected cells stably expressing the Flag-tagged eIF-3f. a. IBV infection of Vero cell clones stably expressing of the Flag-tagged eIF3f. Two stable Vero cell clones with high level expression of the Flag-tagged eIF3f (lanes 2, 4, 6, 8 and 10) and undetectable expression of the Flag-tagged eIF3f (lanes 1, 3, 5, 7 and 9), respectively, were infected with IBV at a multiplicity of infection of 2 and harvested at 0, 8, 12, 16 and 24 hours post-infection. The expression of the Flag-tagged eIF3f was analyzed by Western blot with anti-Flag antibody. The same membrane was also probed with anti-actin antibodies as a loading control. b. Analysis of viral RNA replication and protein synthesis in IBV-infected stable cells clones with high expression of eIF3f (+eIF3f) (lanes 1–5) and undetectable expression of eIF3f (-eIF3f) (lanes 6–10). The two stable Vero cell clones were infected with IBV at a multiplicity of infection of 2 and harvested at 0, 8, 12, 16 and 24 hours post-infection. Ten micrograms of total RNA extracted from one portion of the harvested infected cells were separated on 1.3% agarose gel and transferred to a Hybond N+ membrane. Viral RNAs were probed with a Dig-labeled DNA probe corresponding to the 3′ end 680 nucleotides of the IBV genome. Bands corresponding to the genomic and subgenomic mRNA are indicated by numbers 1–6 on the right. The same membrane was also probed with a Dig-labeled GAPDH probe as loading control. The viral protein expression was analyzed by Western blot of total cell lysates prepared from another portion of the harvested infected cells with anti-IBV S protein antibodies. The same membrane was also analyzed by Western blot with anti-actin antibodies. c. Analysis of IL-8 expression. The total RNA preparations were analyzed by Northern blot with a Dig-labelled IL-8 probe (upper panels). The IL-8 expression at the protein level was analyzed by Western blot analysis with anti-IL-8 antibodies (lower panels). d. Analysis of IL-6 expression. The total RNA preparations were analyzed by Northern blot with a Dig-labelled IL-6 probe (lanes 1–8). The IL-6 expression at the protein level was analyzed by Western blot analysis of culture media (lanes 9 and 10) and total cell lysates (lanes 11–12) harvested 24 h post-infection with anti-IL-6 antibodies. Western blot analysis of the total cell lysates with anti-actin antibodies was included as a loading control (lanes 11 and 12).

A stable cell line expressing the Flag-tagged eIF3f was then established from Vero cells to further study the inhibitory effect of S protein on the expression of IBV-induced genes. Several G418-selected clones showed the expression of the Flag-tagged eIF3f with different efficiencies. One stable cell line with high expression and one with undetectable expression of the Flag-eIF3f were chosen for the study (Fig. 6a). The two cell clones were infected with IBV in a time-course experiment (Fig. 6a). Analysis of viral RNA at 0, 8 12, 16 and 24 hours post-infection, respectively, showed the presence of very similar amounts of viral RNAs in both cell clones (Fig. 6b). However, significantly less S protein was detected in cells stably expressing the Flag-tagged eIF3f at earlier time points (Fig. 6b, lanes 3, 4, 8 and 9). Quantitative analysis of the S protein bands by densitometry showed that 3.87-, 4.65- and 0.9-fold more S protein was detected in cells without expressing the Flag-tagged eIF3f than that in cells expressing the Flag-tagged eIF3f at 12, 16 and 24 hours post-infection, respectively (Fig. 6b, lanes 3–5 and 8–10).

Analysis of IL-8 at the transcriptional level by Northern blot showed induction of IL-8 mRNA in both cell clones infected with IBV, but obviously more induction of IL-8 mRNA in cells without expressing the Flag-tagged eIF-3f was detected (Fig. 6c, upper panels). Western blot analysis of IL-8 at the translational level showed detection of IL-8 in both cell clones (Fig. 6c, lower panels). Interestingly, much more induction of the IL-8 protein expression was detected in cells expressing the Flag-tagged eIF3f at earlier time points (Fig. 6c, lower panels). Quantitative analysis by densitometry showed that 6-, 2.58- and 0.82-fold more IL-8 was present in cells expressing the Flag-tagged eIF3f than that in cells without expressing the Flag-tagged eIF3f at 12, 16 and 24 hours post-infection, respectively (Fig. 6c, lanes 3–5 and 8–10). These results confirm that stable expression of the Flag-tagged eIF3f could offset the inhibitory effect of S protein on the translation of virus-inducible genes.

Similarly, analysis of IL-6 at the transcriptional level by Northern blot showed induction of IL-6 mRNA in both cell clones infected with IBV at 8 (Fig. 6d, lanes 1 and 5), 12 (Fig. 6d, lanes 2 and 6), 16 (Fig. 6d, lanes 3 and 7) and 24 (Fig. 6d, lanes 4 and 8) hours post-infection, respectively. Slightly more induction (1.02-fold) of IL-6 mRNA in cells without expressing the Flag-tagged eIF-3f was observed at 16 hour post-infection (Fig. 6d, lanes 3 and 7). The expression of IL-6 at the translational level was then analyzed by collecting the culture media and total infected cells at 24 hours post-infection by Western blot. Drastically increased detection (5-fold) of IL-6 in the supernatants in cells expressing the Flag-tagged eIF3f was detected (Fig. 6d, lanes 9 and 10). Slightly enhanced expression of IL-6 was also detected in total cell lysates (Fig. 6d, lanes 11 and 12).

## Discussion

Induction and over-production of proinflammatory cytokines and chemokines, such as IL-6, IL-8, TNF-α and INF-γ, were considered to be main mediators in the pathogenesis of SARS [5], [8]–[9]. These cytokines and chemokines could promote inflammation by induction of acute cell and tissue injury. Understanding the cellular and viral mechanisms that regulate the induction of these molecules at the transcriptional and translational levels would be essential for understanding the pathogenesis of coronavirus. In this study, the S protein of SARS-CoV and IBV was shown to interact with the initiation factor eIF-3f, leading to the inhibition of general protein synthesis. As the inhibition occurs at late stages of the virus replication cycle, the main effect would be on the translation of virus-induced transcripts, including predominantly proinflammatory cytokines and chemokines. This inhibition may therefore play an important role in regulation of the pathogenesis of coronavirus.

As an illustrative example shown in this study, IBV-infection of Vero cells induces a drastic increase of IL-6 mRNA transcription at 8–24 hours post-infection. However, only a moderate increase of IL-6 expression at the protein level was detected. The same phenomena were also reported in cells infected with SARS-CoV and in SARS patients. The IL-6 mRNA was significantly up-regulated in SARS-CoV-infected dendritic cells [5]. However, only slight increase of IL-6 at the protein level was detected in the serum of SARS patients [9]. In addition, Tseng et al. [34] also reported that IL-6 protein only increased two fold in macrophages and dendritic cells infected SARS-CoV. These studies support that differential regulation of host cell mRNA transcription and protein synthesis occurs in coronavirus-infected cells. The inhibitory effect on translation rendered by the interaction between coronavirus S protein and eIF3f may account for the observed differential induction of virus-inducible genes at the transcriptional and translational levels.

Is the observed inhibition of translation by the interaction of coronavirus S protein with eIF3f beneficial to the virus? The inhibitory effect appears to be general, as the synthesis of both viral and cellular proteins can be affected. However, as accumulation of the S protein to a certain level in the virus-infected cells is a prerequisite for the inhibition, it would be effective only at late stages of viral infection. This would be largely beneficial to the virus. At this stage, the viral RNA replication and protein synthesis in a given infected cell are nearly complete, inhibition of viral protein synthesis would render little effect on viral production and subsequent infection of neighboring cells. On the contrary, inhibition of host protein synthesis, especially the translation of virus-induced transcripts which are accumulated at very high concentration at this stage, would dramatically reduce the production of protein products from these genes. As most of these products are proinflammatory cytokines and chemokines, synthesis and secretion of these products would cause inflammatory response, sensitize the neighboring cells as well as causing cell death. The most direct consequence of these cell/tissue responses would be the limitation and elimination of viral infection. This study therefore reveals a novel viral mechanism that controls the cellular anti-viral response. In fact, infection of cells stably expressing the Flag-tagged eIF3f with IBV showed reduced virus production, compared with cells without expression of the protein. It supports that inhibition of the translation of virus-induced transcripts is beneficial to the virus. At present, we could not rule out the possibility that stable expression of the Flag-tagged eIF3f may affect the general translation of host cells, although the cells appear to be normal.

Attempts to map the interaction region in the S protein in more details by co-immunoprecipitation and yeast two-hybrid system were unsuccessful, due to the presence of multiple interaction sites. Sequence comparison between the SARS-CoV S protein and the IBV S protein showed low homology (26.1% amino acid identity). However, regions with high similarity in the amino acid sequences between the two S proteins were found. For example, the region from amino acids 207–264 of the IBV S protein shares 58.62% amino acid similarity with the region from amino acids 264–321 in the SARS-CoV S protein. Alternatively, the interaction would be mediated by the carbohydrate chain of the S protein. However, as no other glycoproteins of coronavirus were found to be interacted with eIF3f, there is no evidence to support this speculation at present.

Where is the precise cellular site for this interaction? Coronavirus S protein is a type I membrane protein. The N-terminal part of the protein is normally not exposed to the cytoplasmic side of the membranous compartments of the cell. On the other hand, eIF3f is presumably a protein located mainly in the cytosol and nucleus [35]–[36]. This would prevent or at least reduce the contact between the two proteins. However, immunofluorescent staining of cells expressing the two proteins clearly showed that the SARS-CoV S protein could interact with eIF3f and sequester the later to the site of S protein. It is likely that eIF3f could gain access to the luminal side of the membranous compartments and interact with the S protein. An alternative explanation may lie on the fact that multiple N-terminally truncated forms of the S protein can be detected in cells either infected with SARS-CoV and IBV or transfected with S protein constructs from both viruses (data not shown). As no transmembrane domain in this region, these N-terminally truncated S species may be localized mainly in the cytoplasm and interact with eIF3f.

Cell-cell fusion can be induced in cells either infected with most coronaviruses or transfected with the S protein construct. Interestingly, it was consistently observed that a higher level of S protein expression was detected in cells expressing fusion-competent IBV S protein constructs than that in cells expressing fusion incompetent IBV S protein (data not shown). Similar observations were also reported in studies of other coronavirus S proteins [37]–[39]. Fusion of the neighboring cells would provide additional fresh eIF3f, which in turn enhances the expression of virus-induced transcripts pre-existed in the originally infected cells. It is therefore likely that inhibition of S protein-mediated fusion may, in addition to the inhibition of virus entry, alleviate the production of pathological cytokines and chemokines, leading to the reduction of cell damage caused by these products during the course of coronavirus infection. This would open a new area for design of anti-viral therapeutics.

## References

[1] Cebulla CM, Miller DM, Sedmak DD (1999). Viral inhibition of interferon signal transduction.. Intervirology.

[2] Cheung CY, Poon LL, Lau AS, Luk W, Lau YL (2002). Induction of proinflammatory cytokines in human macrophages by influenza A (H5N1) viruses: a mechanism for the unusual severity of human disease?. Lancet.

[3] Guidotti LG, Chisari FV (2000). Cytokine-mediated control of viral infections.. Virology.

[4] Jones BM, Ma ES, Peiris JS, Wong PC, Ho JC (2004). Prolonged disturbances of in vitro cytokine production in patients with severe acute respiratory syndrome (SARS) treated with ribavirin and steroids.. Clin Exp Immunol.

[5] Law HKW, Cheung CY, Ng HY, Sia SF, Chan YO (2005). Chemokine up-regulation in SARS-coronavirus–infected, monocyte-derived human dendritic cells.. Immunobiology..

[6] Yu LM, Hui DS, Tam JS, Cheng G, Sung JJ (2003). Haematological manifestations in patients with severe acute respiratory syndrome: retrospective analysis.. British Medical Journal.

[7] Glass WG, Subbarao K, Murphy B, Murphy PM (2004). Mechanisms of host defense following severe acute respiratory syndrome-coronavirus (SARS-CoV) pulmonary infection of mice.. J Immunol.

[8] Wong CK, Lam CW, Wu AK, Ip WK, Lee NL (2004). Plasma inflammatory cytokines and chemokines in severe acute respiratory syndrome.. Clin Exp Immunol.

[9] Zhang YC, Li J, Zhan YL, Wu LQ, Yu X (2004). Analysis of Serum Cytokines in Patients with Severe Acute Respiratory Syndrome.. Infection and Immunity.

[10] Ziegler T, Sampsa M, Esa R, Pamela O, Maarit S (2005). Severe acute respiratory syndrome coronavirus fails to activate cytokine-mediated innate immune responses in cultured human monocyte-derived dendritic cells.. J Virol.

[11] Peiris JS, Lai ST, Poon LL, Guan Y, Yam LY (2003). Coronavirus as a possible cause of severe acute respiratory syndrome.. Lancet.

[12] Rota PA, Oberste MS, Monroe SS, Nix WA, Campagnoli R (2003). Characterization of a novel coronavirus associated with severe acute respiratory syndrome.. Science.

[13] Marra MA, Jones SJ, Astell CR, Holt RA, Brooks-Wilson A (2003). The genome sequence of the SARS-associated coronavirus.. Science.

[14] Snijder EJ, Bredenbeek PJ, Dobbe JC, Thiel V, Ziebuhr J (2003). Unique and Conserved features of genome and proteome of SARS-coronavirus, an early split-off from the coronavirus group 2 lineage.. J Mol Biol.

[15] Thiel V, Ivanov KA, Putics A, Hertzig T, Schelle B (2003). Mechanisms and enzymes involved in SARS coronavirus genome expression.. J Gen Virol.

[16] Silverman RH, Williams BRG (1999). Translational control perks up.. Nature.

[17] Ahmed M, McKenzie MO, Puckett S, Hojnacki M, Poliquin L (2003). Ability of the matrix protein of vesicular stomatitis virus to suppress beta interferon gene expression is genetically correlated with the inhibition of host RNA and protein synthesis.. J Virol.

[18] Black BL, Lyles DS (1992). Vesicular stomatitis virus matrix protein inhibits host cell-directed transcription of target genes in vivo.. J Virol.

[19] Ferran MC, Lucas-Lenard JM (1997). The vesicular stomatitis virus matrix protein inhibits transcription from the human beta interferon promoter.. J Virol.

[20] Yuan H, Yoza BK, Lyles DS (1998). Inhibition of host RNA polymerase II-dependent transcription by vesicular stomatitis virus results from inactivation of TFIID.. Virology.

[21] Schneider RJ, Hershey JWB (2000). Cold Spring Harbor Laboratory Press.. Translational Regulation. Adenovirus inhibition of cellular protein synthesis and preferential translation of viral mRNAs.

[22] Laurent AM, Madjar JJ, Greco A (1998). Translational control of viral and host protein synthesis during the course of herpes simplex virus type 1 infection: evidence that initiation of translation is the limiting step.. J Gen Virol.

[23] Schneider RJ, Mohr I (2003). Translation initiation and viral tricks.. Trends in Biochemical Sciences.

[24] Alcami A, Koszinowski UH (2000). Viral mechanisms of immune evasion.. Immunol Today.

[25] Ali IK, McKendrick L, Morley SJ, Jackson RJ (2001). Truncated initiation factor eIF4G lacking an eIF4E binding site can support capped mRNA translation.. EMBO J.

[26] Gale MJ, Tan SL, Katze MG (2000). Translational control of viral gene expression in eukaryotes.. Microbiol. Mol Biol Rev.

[27] Joachims M, Van-Breugel PC, Lloyd RE (1999). Cleavage of poly(A)-binding protein by enterovirus proteases concurrent with inhibition of translation in vitro.. J Virol.

[28] Lamphear B, Yan R, Yang F, Waters D, Liebig HD (1993). Mapping the cleavage site in protein synthesis initiation factor eIF-4g of the 2A proteases from human Coxsackievirus and rhinovirus.. J Biol Chem.

[29] Katsura A, Kinzy TG, Merrick WC, Hershey JWB (1997). Conservation and Diversity of Eukaryotic Translation Initiation Factor eIF3.. J Biol Chem.

[30] Keith RJ, Merrick WC, Zoll WL, Zhu Y (1997). Identification of cDNA Clones for the Large Subunit of Eukaryotic Translation Initiation Factor 3. Comparison of homologues from human, nicotiana tabacum, caenorhabditis elegans, and saccharomyces cerevisiae.. J Biol Chem.

[31] Nathalie M, Rom E, Olsen H, Sonenberg N (1997). The Human Homologue of the Yeast Prt1 Protein Is an Integral Part of the Eukaryotic Initiation Factor 3 Complex and Interacts with p170.. J Biol Chem.

[32] Wang L, Tam JP, Liu DX (2006). Biochemical and functional characterization of Epstein-Barr Virus-encoded BARF1 protein: interaction with human hTid1 protein facilitates its maturation and secretion.. Oncogene.

[33] Liu DX, Xu HY, Brown TDK (1997). Proteolytic processing of the coronavirus infectious bronchitis virus 1a polyprotein: Identification of a 10 kDa polypeptide and determination of its cleavage sites.. J Virol.

[34] Tseng CT, Perrone LA, Zhu H, Makino S, Peters CJ (2005). Severe acute respiratory syndrome and the innate immune responses: modulation of effector cell function without productive infection.. J Immunol.

[35] Shi J, Kahle A, Hershey JW, Honchak BM, Warneke JA (2006). Decreased expression of eukaryotic initiation factor 3f deregulates translation and apoptosis in tumor cells.. Oncogene.

[36] Shi J, Feng Y, Goulet AC, Vaillancourt RR, Sachs NA (2003). The p34cdc2-related cyclin-dependent kinase 11 interacts with the p47 subunit of eukaryotic initiation factor 3 during apoptosis.. J. Biol. Chem..

[37] Bos EC, Heijnen L, Luytjes W, Spaan WJ (1995). Mutational analysis of the murine coronavirus spike protein: effect on cell-to-cell fusion.. Virology.

[38] Bosch BJ, van der Zee R, de Haan CAM, Rottier PJM (2003). The coronavirus spike protein is a class I virus fusion protein: structural and functional characterization of the fusion core complex.. J Virol.

[39] de Haan CAM, Stadler K, Godeke GJ, Bosch BJ, Rottier PJM (2004). Cleavage inhibition of the murine coronavirus spike protein by a furin-like enzyme affects cell-cell but not virus-cell fusion.. J Virol.

